# Clinicopathologic significance of sialyl Le ^x^expression in advanced gastric carcinoma

**DOI:** 10.1054/bjoc.2000.1484

**Published:** 2000-12

**Authors:** N Futamura, S Nakamura, M Tatematsu, Y Yamamura, R Kannagi, H Hirose

**Affiliations:** First Department of Surgery, Gifu University School of Medicine; Department of Pathology and Clinical Laboratories, Aichi Cancer Center Hospital; Laboratory of Pathology I, Aichi Cancer Center Research Institute; Department of Gastroenterological Surgery, Aichi Cancer Center Hospital; Laboratory of Pathology II, Aichi Cancer Center Research Institute

**Keywords:** gastric carcinoma, sialyl Le ^x^, SNH-3, immunohistochemistry

## Abstract

Sialyl Lewis ^x^antigen (SLX) is a carbohydrate antigen that serves as a ligand for selectin, an adhesion molecule expressed on vascular endothelial cells. The expression of SLX in 245 patients with advanced gastric carcinoma was examined immunohistochemically, and its clinicopathologic significance was analysed. We classified the patients with advanced gastric carcinoma into 91 with differentiated type and 154 with undifferentiated type. SLX expressed in 135 of 245 patients (55%), comprising 68 (75%) patients with differentiated carcinoma and 67 (44%) with undifferentiated carcinoma. The positive rate for SLX expression was significantly higher among patients with differentiated carcinoma than among those in undifferentiated carcinoma (*P* < 0.0001). With differentiated carcinoma, the incidence of lymph node metastasis, advanced tumour stage (stage III and IV) and liver recurrence was significantly higher in SLX-positive patients than in SLX-negative ones (*P*  < .0001, *P* = 0.0065 and *P* = 0.028, respectively). Moreover, the prognoses were better in patients with SLX-negative tumours than in those with SLX-positive tumours (*P* = 0.019). With undifferentiated carcinoma, there were no significant correlations between SLX expression and any clinicopathological features or prognoses. The clinicopathologic significance of SLX expression in gastric carcinoma patients depends on histologic type. SLX expression may be of great relevance in predicting liver metastases in patients with differentiated carcinoma. © 2000 Cancer Research Campaign http://www.bjcancer.com


*British Journal of Cancer* (2000) **83**(12), 1681–1687
© 2000 Cancer Research Campaign
doi: 10.1054/ bjoc.2000.1484, available online at http://www.idealibrary.com on IDEAL®

### Clinicopathologic significance of sialyl Le$^x$ expression in advanced gastric carcinoma

**N Futamura[1], S Nakamura[2], M Tatematsu[3], Y Yamamura[4], R Kannagi[5] and H Hirose[1]**

[1]First Department of Surgery, Gifu University School of Medicine; [2]Department of Pathology and Clinical Laboratories, Aichi Cancer Center Hospital; [3]Laboratory of Pathology I, Aichi Cancer Center Research Institute; [4]Department of Gastroenterological Surgery, Aichi Cancer Center Hospital; [5]Laboratory of Pathology II, Aichi Cancer Center Research Institute

**Summary** Sialyl Lewis$^x$ antigen (SLX) is a carbohydrate antigen that serves as a ligand for selectin, an adhesion molecule expressed on vascular endothelial cells. The expression of SLX in 245 patients with advanced gastric carcinoma was examined immunohistochemically, and its clinicopathologic significance was analysed. We classified the patients with advanced gastric carcinoma into 91 with differentiated type and 154 with undifferentiated type. SLX expressed in 135 of 245 patients (55%), comprising 68 (75%) patients with differentiated carcinoma and 67 (44%) with undifferentiated carcinoma. The positive rate for SLX expression was significantly higher among patients with differentiated carcinoma than among those in undifferentiated carcinoma ($P < 0.0001$). With differentiated carcinoma, the incidence of lymph node metastasis, advanced tumour stage (stage III and IV) and liver recurrence was significantly higher in SLX-positive patients than in SLX-negative ones ($P < 0.0001$, $P = 0.0065$ and $P = 0.028$, respectively). Moreover, the prognoses were better in patients with SLX-negative tumours than in those with SLX-positive tumours ($P = 0.019$). With undifferentiated carcinoma, there were no significant correlations between SLX expression and any clinicopathological features or prognoses. The clinicopathologic significance of SLX expression in gastric carcinoma patients depends on histologic type. SLX expression may be of great relevance in predicting liver metastases in patients with differentiated carcinoma. © 2000 Cancer Research Campaign http://www.bjcancer.com

**Keyword**: gastric carcinoma; sialyl Le$^x$; SNH-3; immunohistochemistry

The incidence of gastric carcinoma is high among Japanese. The recent development of early diagnostic methods has improved the prognoses of patients with gastric carcinoma. However, prognoses remain unsatisfactory in patients with advanced gastric carcinoma.

There are many histological classifications of gastric carcinoma. Gastric carcinomas were classified into the intestinal type and the diffuse type by Lauren (1965), and into papillary adenocarcinoma (PAP), tubular adenocarcinoma (TUB), poorly differentiated adenocarcinoma (POR), signet-ring cell carcinoma (SIG) and mucinous adenocarcinoma (MUC) in the Japanese Classification of Gastric Carcinoma (1995). To evaluate the biological role of sialyl Lewis$^x$ antigen (SLX) depending on histologic subtype in the present study, we classified gastric carcinomas into differentiated type and undifferentiated type as follows: differentiated carcinoma consisted of PAP, TUB and MUC which arose from PAP or TUB, and undifferentiated carcinoma of POR, SIG and MUC which arose from POR or SIG. Differentiated carcinoma corresponds to the intestinal type according to Lauren, and undifferentiated carcinoma to the diffuse type. The proportions of both men and older patients are greater among the differentiated carcinoma patients than the undifferentiated carcinoma patients (Lauren, 1965; Wronkowsky et al, 1977). With respect to the metastatic pattern, differentiated carcinoma tends to metastasize to the liver, and undifferentiated carcinoma to the peritoneum (Ignacio and Osvaldo, 1981; Rhomberg and Gruber, 1989; Esaki et al, 1990; Mori et al, 1995).

Carbohydrate antigens including SLX and sialyl Lewis$^a$ antigen have been used as tumour markers of many carcinomas, including gastric carcinoma. It was reported that these carbohydrate antigens are ligands for selectin, an adhesion molecule expressed on the vascular endothelial cells (Lowe et al, 1990; Phillips et al, 1990; Walz et al, 1990; Takada et al, 1991). In vitro study showed that these carbohydrates were involved in the adhesion of tumour cells from gastric carcinoma, colon carcinoma, hepatoma, pancreatic carcinoma and lung carcinoma to E-selectin, which is present on cytokine-activated human endothelial cells (Takada et al, 1993). It was also reported that colon carcinoma cells which were highly metastatic to the liver expressed relatively more SLX structures than their corresponding low metastatic counterparts, and that the increased expression of SLX correlated with strong adherence to vascular endothelial cells with E-selectin (Saitoh et al, 1992; Sawada et al, 1992; Izumi et al, 1995; Bresalier et al, 1996). These findings suggest that SLX play important roles during haematogeneous tumour metastasis. However, regarding the mechanism of peritoneal dissemination, Nakashio et al (1997) suggested that SLX was not involved in peritoneal dissemination.

In recent years, correlations between the expression of these carbohydrates and outcomes have been reported for various tumours, although some studies have yielded conflicting results (Nakagoe et al, 1993; Nakamori et al, 1993, 1997; Narita et al, 1993; Ogawa et al, 1994; Jorgensen et al, 1995; Nakayama et al, 1995; Amado et al, 1998). Information regarding the significance

*Received 14 January 2000*
*Revised 5 July 2000*
*Accepted 14 August 2000*

*Correspondence to:* Naoki Futamura of SLX expression in gastric carcinoma is limited (Nakamori et al, 1997; Ura et al, 1997; Amado et al, 1998). These studies did not satisfactorily mention the differences based on the histologic types.

In the present study, we evaluated the clinicopathologic significance of SLX in patients with advanced gastric carcinoma, who had undergone gastric resections between January, 1980 and December, 1981. We classified gastric carcinomas into differentiated type, in which liver metastasis was more common, and undifferentiated type, which had more frequent peritoneal dissemination. Based on the hypothesis that the influence of SLX expression may have been obscured by distinct biological roles depending on histologic subtypes, we also analysed the clinicopathologic significance of SLX expression in subsets of patients with differentiated and undifferentiated carcinoma.

### MATERIALS AND METHODS

### Patients

We studied 245 patients with advanced gastric carcinoma who underwent consecutive gastrectomies at Aichi Cancer Center Hospital between January, 1980 and December, 1981. They were followed well for 10 years. Histologically, they were divided into 91 patients with differentiated carcinoma and 154 with undifferentiated carcinoma. Tumour stage and curative potential of the gastric resection were classified by the Japanese Classification of Gastric Carcinoma (1995). In this study, tumour stages III and IV were regarded as advanced tumour stages. Lymphadenectomy was performed on 242 patients.

The proportions of men and older patients were greater among cases of differentiated than undifferentiated carcinomas ($P < 0.0001$ and $P < 0.0001$, respectively). Moreover, the incidence of invasion to the serous membrane was significantly lower with differentiated carcinoma than with undifferentiated carcinoma ($P = 0.0002$). The incidence of synchronous liver metastases at surgery was significantly higher ($P = 0.021$) among differentiated than among undifferentiated carcinoma patients, while the incidence of synchronous peritoneal dissemination at surgery was significantly lower ($P = 0.023$) among differentiated than among undifferentiated carcinoma patients (Table 1). Gastric resections for 198 patients were regarded as potentially curative (Resection A and Resection B according to the Japanese Classification of Gastric Cancer (1995)), and for 47 patients as noncurative (Resection C according to the Japanese Classification of Gastric Cancer (1995)). Of 198 patients who underwent potentially curative resection, 91 patients died of a recurrence of the gastric carcinoma and 20 of some other disease.

### Antibody

Immunostaining was performed using SNH-3, a monoclonal antibody against SLX (IgM, kindly supplied by Dr Sen-itiroh Hakomori, Pacific Northwest Research Foundation, Seattle, WA, USA).

### Immunohistochemical staining and evaluation

The tissue specimens were fixed in 10% neutral-buffered formaldehyde solution, embedded in paraffin, and cut into 5 μm sections. An avidin-biotin complex technique was used. Each reaction was carried out at room temperature. In brief, endogenous peroxidase activity was blocked by 0.3% hydrogen peroxide in methanol. Tissue specimens were reacted with SNH-3 antibody for 1 hour in a moist chamber. Subsequently, tissue specimens were reacted with anti-mouse IgG, avidin-biotin complex (Vectastatin ABC kit, Vecter, Burlingame, CA), and 3,3′-diaminobenzidine tetrahydrochloride. The specimens were counterstained with haematoxylin. The stained tissue specimens were evaluated by 2 of the authors. Based on the ratio of SLX-positive cells, the positivity of the tumour cells was classified as follows:

(–): less than 10% of tumour cells were positive for SLX;

(+): 10% or more of tumour cells were positive for SLX.

### Staining of elastic fibres

To evaluate the presence of venous invasion, Victoria blue stain (Tsutsumi et al, 1990) was used to stain elastic fibres.

**Table 1** Cinicopathological features of 245 patients with advanced gastric carcinoma

| | Total $n$ = 245 (%) | Differentiated carcinoma $n$ = 91 (%) | Undifferentiated carcinoma $n$ = 154 (%) | $P$ value |
|---|---|---|---|---|
| Average age | 56.9 ± 12.0 | 63.4 ± 9.5 | 53.1 ± 12.0 | $P < 0.0001$ |
| Sex | | | | |
| Male | 163 (67) | 76 (84) | 87 (56) | $P < 0.0001$ |
| Female | 82 (33) | 15 (16) | 67 (44) | |
| Serosal invasion | | | | |
| Positive | 136 (56) | 36 (40) | 100 (65) | $P = 0.0002$ |
| Lymph node metastasis | | | | |
| Positive | 173/242 (71) | 1/90 (68) | 112/152 (74) | $P = 0.40$ |
| Venous invasion | | | | |
| Positive | 118 (48) | 49 (54) | 69 (45) | $P = 0.22$ |
| Liver metastasis | | | | |
| Positive | 7 (3) | 6 (7) | 1 (0.6) | $P = 0.021$ |
| Peritoneum dissemination | | | | |
| Positive | 30 (12) | 5 (5) | 25 (16) | $P = 0.023$ |
| Stage | | | | |
| I (I a + I b) | 48 (20) | 22 (24) | 26 (17) | $P = 0.11$ |
| II | 45 (18) | 21 (23) | 24 (16) | |
| III (IIIa + IIIb) | 97 (40) | 33 (36) | 64 (42) | |
| IV (IVa + IVb) | 55 (22) | 15 (16) | 40 (26) | |

 *© 2000 Cancer Research Campaign*

### Statistical analysis

Respective factors were compared by the $\chi^2$ test or Student's *t*-test. Survival rates were calculated by the Kaplan-Meier method, and statistical significance was tested with a generalized Wilcoxon test.

### RESULTS

### Relationship between recurrent site and histologic types

Of the 79 patients with differentiated carcinoma who underwent potentially curative resection, 34 patients died of gastric carcinoma recurrence and 11 of other diseases. The initial diagnoses of recurrent tumors were as follows: liver metastasis in 13 (38%) patients, peritoneal dissemination in 11 (32%) patients, and recurrent tumour from other sites in 10 (29%) patients. Of the 119 patients with undifferentiated carcinoma who underwent potentially curative resection, 57 patients died of a recurrence of gastric carcinoma and 9 of other diseases. The initial diagnoses of recurrent tumours were as follows: peritoneal dissemination in 37 (65%) patients, liver metastasis in one (1%) patient, and recurrent tumour from other sites in 19 (33%) patients. Statistical analysis revealed that the incidence of recurrence in the liver was significantly higher ($P < 0.0001$) among patients with differentiated carcinoma than among those with undifferentiated carcinoma, while the incidence of recurrence in the peritoneum was significantly higher ($P < 0.0096$) among patients with undifferentiated carcinoma than among those with differentiated carcinoma (Table 2).

### Expression of SLX

In cancerous gastric tissue, positive staining for SLX was mainly observed in cell membranes and cytoplasm (Figure 1). Positive SLX staining was sometimes obtained in the extracellular mucus.

### Relationship between SLX expression and clinicopathologic features in patients with advanced gastric carcinoma

The correlations of SLX expression with different clinicopathologic features are shown in Table 3. Among 245 patients with advanced gastric carcinoma, SLX was positive in 135 (55%) patients, including 68 (75%) with differentiated carcinoma and 67 (44%) with undifferentiated carcinoma. The rate of positive SLX expression was significantly higher among differentiated carcinoma patients than among undifferentiated carcinoma patients ($P < 0.0001$). The incidence of differentiated carcinoma, venous invasion and lymph node metastasis was significantly higher ($P < 0.0001$, $P = 0.016$ and $P = 0.0037$, respectively) in SLX-positive patients than in SLX-negative ones. No significant correlations were found between SLX expression and the other clinicopathologic features.

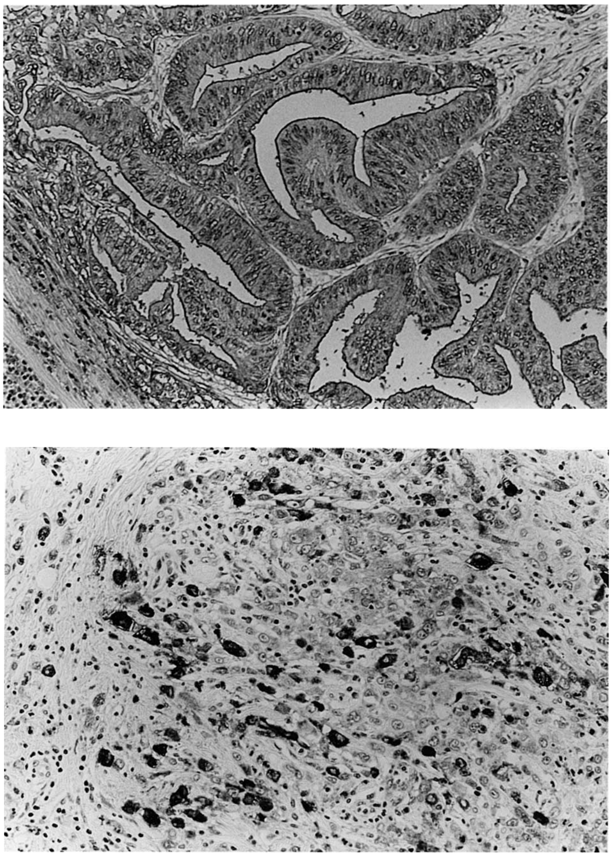

**Figure 1** In cancerous gastric tissue, SLX expression was located mainly on the cell membrane and in the cytoplasm. (**A**) differentiated carcinoma, (**B**) undifferentiated carcinoma (original magnification ×100)

### Relationship between SLX expression and clinicopathologic features in patients with differentiated carcinoma

The correlations of SLX expression with different clinicopathologic features are shown in Table 4. SLX was positive in 68 (75%) of 91 patients with differentiated carcinoma. The incidence of lymph node metastasis and advanced tumour stage were significantly higher ($P < 0.0001$ and $P = 0.0065$, respectively) in SLX-positive patients than in SLX-negative ones. No significant correlations were found between SLX expression and the other clinicopathologic features. The 6 patients with synchronous liver metastasis at surgery were all positive for SLX.

### Relationship between SLX expression and clinicopathologic features in patients with undifferentiated carcinoma

The correlations of SLX expression with different clinicopathologic features are shown in Table 5. SLX was positive in 67 (44%)

**Table 2** Postoperative liver metastasis and peritoneal dissemination in patients with curative resoction

| | Total *n* = 198 (%) | Differentiated carcinoma *n* = 79 (%) | Undifferentiated carcinoma *n* = 119 (%) | *P* value |
|---|---|---|---|---|
| Liver metastasis | 14 (7) | 13 (16) | 1 (1) | $P < 0.0001$ |
| Peritoneal dissemination | 48 (24) | 11 (14) | 37 (31) | $P = 0.0096$ |

© 2000 Cancer Research Campaign 

**Table 3** Relationship between SLX staining status and clinicopathologic features in patients with advanced gastric carcinoma

| | SLX (–) *n* = 110 (%) | SLX (+) *n* = 135 (%) | *P* value |
|---|---|---|---|
| Age | | | |
| ≥57 | 52 (47) | 72 (53) | *P* = 0.41 |
| 57> | 58 (53) | 63 (47) | |
| Sex | | | |
| Male | 69 (63) | 94 (70) | *P* = 0.24 |
| Female | 41 (37) | 41 (30) | |
| Histologic type | | | |
| Differentiated type | 23 (21) | 68 (50) | *P* < 0.0001 |
| Undifferentiated type | 87 (79) | 67 (50) | |
| Serosal invasion | | | |
| Positive | 64 (58) | 71 (53) | *P* = 0.46 |
| Venous invasion | | | |
| Positive | 44 (40) | 76 (56) | *P* = 0.016 |
| Lymph node metastasis | | | |
| Positive | 68 (62) | 105/132 (80) | *P* = 0.0037 |
| Liver metastasis | | | |
| Positive | 1 (0.9) | 6 (4) | *P* = 0.21 |
| Peritoneum dissemination | | | |
| Positive | 15 (14) | 15 (11) | *P* = 0.69 |
| Stage | | | |
| I + II | 48 (44) | 45 (33) | *P* = 0.13 |
| III + IV | 62 (56) | 90 (67) | |
| Curability of resection | | | |
| Potentially curative | 88 (80) | 110 (71) | *P* = 0.90 |
| Noncurative | 22 (20) | 2 5(19) | |

**Table 4** Relationship between SLX staining status and clinicopathologic features in patients with differentiated carcinoma

| | SLX (–) *n* = 23 (%) | SLX (+) *n* = 68 (%) | *P* value |
|---|---|---|---|
| Age | | | |
| ≥65 | 16 (70) | 31 (46) | *P* = 0.08 |
| 65> | 7 (30) | 37 (54) | |
| Sex | | | |
| Male | 21 (91) | 55 (81) | *P* = 0.40 |
| Female | 2 (9) | 13 (19) | |
| Serosal invasion | | | |
| Positive | 6 (26) | 30 (44) | *P* = 0.24 |
| Venous invasion | | | |
| Positive | 10 (43) | 39 (57) | *P* = 0.36 |
| Lymph node metastasis | | | |
| Positive | 7 (30) | 54/67 (81) | *P* < 0.0001 |
| Liver metastasis | | | |
| Positive | 0 | 6 (9) | *P* = 0.32 |
| Peritoneal dissemination | | | |
| Positive | 0 | 5 (7) | *P* = 0.42 |
| Stage | | | |
| I + II | 17 (74) | 26 (38) | *P* = 0.0065 |
| III + IV | 6 (26) | 42 (62) | |
| Curability of resection | | | |
| Potentially curative | 23 (100) | 56 (82) | *P* = 0.07 |
| Noncurative | 0 | 12 (18) | |

of 154 patients with undifferentiated carcinoma. There was no significant difference between SLX expression and any clinicopathologic features.

### Relationship between SLX expression and survival

In survival analysis with advanced gastric carcinoma ($n = 245$), there was no significant difference between SLX expression and survival (Figure 2). 44 (49%) of 110 SLX-negative patients and 84 (62%) of 135 SLX-positive ones died of gastric carcinoma, indicating no significant difference in mortality from gastric carcinoma between SLX-negative and SLX-positive patients.

With differentiated carcinoma ($n = 91$), the prognoses of SLX-positive patients were significantly poorer than those of SLX-negative patients ($P = 0.019$, Figure 3). 4 (17%) of 23 SLX-negative patients and 42 (62%) of 68 SLX-positive ones died of gastric carcinoma, indicating that mortality from gastric carcinoma was significantly higher in the latter than in the former ($P = 0.0006$).

 © 2000 Cancer Research Campaign

**Table 5** Relationship between SLX staining status and clinicopathologic features in patients with undifferentiated carcinoma

| | SLX (–) *n* = 87 (%) | SLX (+) *n* = 67 (%) | *P* value |
|---|---|---|---|
| Age | | | |
| ≥54 | 40 (46) | 39 (58) | *P* = 0.18 |
| 54> | 47 (54) | 28 (42) | |
| Sex | | | |
| Male | 48 (55) | 39 (58) | *P* = 0.83 |
| Female | 39 (45) | 28 (42) | |
| Serosal invasion | | | |
| Positive | 58 (67) | 42 (63) | *P* = 0.73 |
| Venous invasion | | | |
| Positive | 34 (39) | 37 (55) | *P* = 0.067 |
| Lymph node metastasis | | | |
| Positive | 61 (70) | 51/65 (78) | *P* = 0.33 |
| Liver metastasis | | | |
| Positive | 1 (1) | 0 | *P* = 0.90 |
| Peritoneal dissemination | | | |
| Positive | 15 (7) | 10 (15) | *P* = 0.87 |
| Stage | | | |
| I + II | 31 (36) | 19 (28) | *P* = 0.43 |
| III + IV | 56 (64) | 48 (72) | |
| Curability of resection | | | |
| Potentially curative | 65 (75) | 54 (81) | *P* = 0.50 |
| Noncurative | 22 (25) | 13 (19) | |

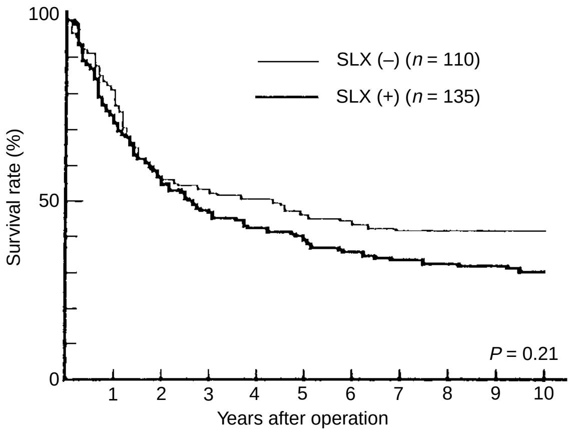

**Figure 2** Survival curves of patients with advanced gastric carcinoma, subdivided according to SLX antigen expression

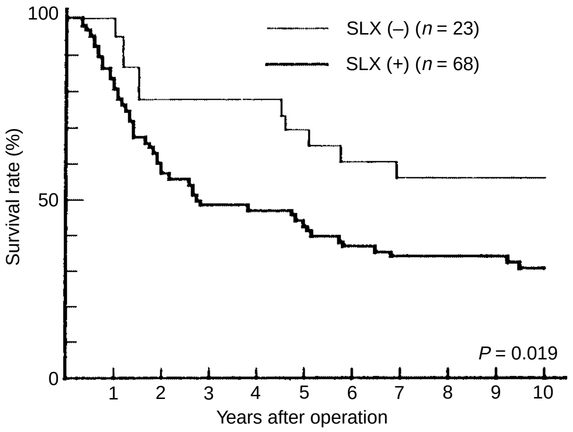

**Figure 3** Survival curves of patients with differentiated carcinoma, subdivided according to SLX antigen expression

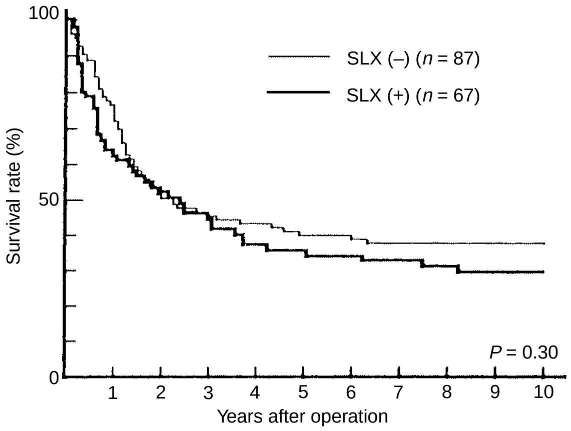

**Figure 4** Survival curves of patients with undifferentiated carcinoma, subdivided according to SLX antigen expression

With undifferentiated carcinoma ($n$ = 154), there was no significant difference between SLX expression and survival (Figure 4). 50 (57%) of 87 SLX-negative patients and 42 (63%) of 67 SLX-positive ones died of gastric carcinoma, indicating no significant difference in mortality from gastric carcinoma between SLX-negative and SLX-positive patients.

### Evaluation of SLX expression in patients who underwent potentially curative resection and developed recurrent tumour in the liver or in the peritoneum

Among 198 patients with advanced gastric carcinoma who underwent potentially curative resection, the incidence of recurrence in the liver was significantly higher in SLX-positive patients than in SLX-negative ones ($P$ = 0.0084). However, there was no

© 2000 Cancer Research Campaign

**Table 6** Relationship between SLX staining status and postoperative liver metastasis and peritoneal dissemination in patients with curative resection

| (a) Advanced gastric carcinoma | SLX(–) *n* = 88 (%) | SLX (+) *n* = 110 (%) | *P* value |
|---|---|---|---|
| Liver metastasis | 1 (1) | 13 (23) | *P* = 0.0084 |
| Peritoneal dissemination | 21 (24) | 27 (25) | *P* = 0.96 |

| (b) Differentiated carcinoma | SLX (–) *n* = 23 (%) | SLX(+) *n* = 56 (%) | *P* value |
|---|---|---|---|
| Liver metastasis | 0 | 13 (23) | *P* = 0.028 |
| Peritoneal dissemination | 3 (13) | 8 (14) | *P* = 0.83 |

| (c) Undifferentiated carcinoma | SLX (–) *n* = 65 (%) | SLX(+) *n* = 54 (%) | *P* value |
|---|---|---|---|
| Liver metastasis | 1 (2) | 0 | *P* = 0.93 |
| Peritoneal dissemination | 18 (28) | 19 (35) | *P* = 0.50 |

significant difference in the incidence of recurrence in the peritoneum between SLX-positive and SLX-negative patients.

Among the 79 patients with differentiated carcinoma who underwent potentially curative resection, no recurrence in the liver was observed in 23 SLX-negative patients, while recurrence in the liver was observed in 13 (23%) of 56 SLX-positive patients, demonstrating a significantly higher incidence of liver recurrence in SLX-positive patients than in SLX-negative ones ($P = 0.028$). There was no significant difference in the incidence of recurrence in the peritoneum between SLX-negative and SLX-positive patients.

Among the 119 patients with undifferentiated carcinoma who underwent potentially curative resection, there were no significant differences in the incidence of recurrence in the liver or in the peritoneum between SLX-negative and SLX-positive patients (Table 6).

### DISCUSSION

The present study demonstrated that differentiated carcinoma tended to metastasize to the liver and undifferentiated carcinoma to the peritoneum, which was consistent with previous studies (Ignasio and Osvaldo, 1981; Rhomberg and Gruber, 1989; Esaki et al, 1990; Mori et al, 1995).

Previous studies regarding the significance of SLX expression in gastric carcinoma are limited and have yielded conflicting results (Nakamori et al, 1997; Ura et al, 1997; Amado et al, 1998). Nakamori et al (1997) reported that SLX expression in gastric carcinoma was not correlated with any clinicopathologic features. However, Ura et al (1997) reported that SLX expression correlated with increased risk of metastases and poor prognosis in gastric carcinoma, and Amado et al (1998) reported that SLX expression in gastric carcinoma was correlated with venous invasion and poor outcome. These studies did not adequately mention the differences based on the histologic types. The purpose in this study was to determine whether the differentiated and undifferentiated types of gastric carcinoma have different patterns in clinicopathologic significance of SLX expression.

Regarding the relationship between SLX positivity and histologic type, only a few studies have been found in English literatures, and yielded conflicting results (Dohi et al, 1989; Ikeda et al, 1996; Nakamori et al, 1997; Ura et al, 1997; Amado et al, 1998). In the present study, the frequency of SLX positivity was significantly higher among patients with differentiated carcinoma than among those with undifferentiated carcinoma ($P < 0.0001$).

There were distinct differences in the clinicopathologic significance of SLX expression between cases of differentiated and undifferentiated carcinoma. With differentiated carcinoma, there were significant correlations between SLX expression and lymph node metastasis, tumour stage, prognosis and tumour recurrence in the liver. However, with undifferentiated carcinoma, there were no significant correlations between the SLX expression and clinicopathologic features.

The incidence of lymph node metastasis with differentiated carcinoma were 30% and 81% in SLX-negative and SLX-positive patients, respectively, indicating a significant difference between the two ($P < 0.0001$). Although the biological significance of SLX in lymph node metastasis remains unknown, this observation suggests that SLX may play an important role in this type of metastasis especially in cases of differentiated carcinoma.

Notably, with differentiated carcinoma, SLX was positive in all 19 patients with synchronous liver metastasis and liver recurrence. The incidence of liver recurrence was significantly higher in SLX-positive patients than in SLX-negative ones ($P = 0.028$). These results were consistent with the results of previous animal and in vitro reports, which stated that SLX was a ligand carbohydrate of selection and played an important role during haematogenous metastasis (Saitoh et al, 1992; Sawada et al, 1992; Takada et al, 1993; Izumi et al, 1995; Bresalier et al, 1996). The present study revealed that the frequencies of SLX positivity, synchronous liver metastasis and liver recurrence were significantly higher with differentiated carcinoma than with undifferentiated carcinoma ($P < 0.0001$, $P = 0.021$ and $P = 0.028$, respectively). Therefore, this high SLX expression appears to be closely related with the higher frequency of liver metastasis in differentiated carcinoma.

The survival rate of patients with SLX-positive tumours was significantly lower ($P = 0.019$) than that of patients with SLX-negative tumours in cases of differentiated carcinoma. The high incidence of lymph node metastasis and liver metastasis may result in poor outcome of SLX-positive patients with differentiated carcinoma.

Peritoneal dissemination was a main metastatic pattern of undifferentiated carcinomas. The present study showed no significant correlations between SLX expression and peritoneal dissemination in patients with advanced, differentiated or undifferentiated gastric carcinomas. Several reports have recently been published which deal with the mechanism of this dissemination. Using ovarian carcinoma cells, Cannistra et al (1993) reported that CD44H was involved in the adhesion of tumour cells to peritoneal mesothelial cells. Some authors have subsequently described that CD44H

 © 2000 Cancer Research Campaign plays an important role in peritoneal dissemination of a gastric carcinoma (Nishimura et al, 1996; Nakashio et al, 1997). Using a gastric carcinoma cell line which was established from undifferentiated carcinoma (Akiyama et al, 1988) and had a high potential for peritoneal dissemination, Nakashio et al (1997) indicated that both CD44H and $\beta_1$ integrin were involved in peritoneal dissemination. Interestingly, their flow-cytometric analysis indicated that SLX expression was weak in the gastric carcinoma cells with a high potential for peritoneal dissemination, and that peritoneal dissemination was not inhibited by anti-SLX antibody. SLX expression may not influence the development of peritoneal dissemination.

In conclusion, the clinicopathologic significance of SLX expression in gastric carcinoma depended on histologic type. With differentiated carcinoma, differences in SLX expression were associated with lymph node metastasis, tumour stage, liver recurrence and prognosis. Expression of SLX may be of great relevance in predicting the liver metastasis in patients with differentiated carcinoma. But there were no significant correlations between the SLX expression and any clinicopathologic features in patients with undifferentiated carcinoma.

### REFERENCES

Akiyama S, Amo H, Watanabe T, Matsuyama M, Sakamoto J, Imaizumi M, Ichihashi H, Kondo T and Takagi H (1988) Characteristics of three human gastric cell lines. NU-GC-2, NU-GC-3 and NU-GC-4. *Jpn J Surg* **18**: 438–446

Amado M, Carneiro F, Seixas M, Clausen H and Sobrinho-Simoes M (1998) Dimeric sialyl-Le$^x$ expression in gastric carcinoma correlates with venous invasion and poor outcome. *Gastroenterology* **114**: 462–470

Bresalier RS, Ho SB, Schoeppner HL, Kim YS, Sleisenger MH, Brodt P and Byrd JC (1996) Enhanced sialylation of mucin-associated carbohydrate structures in human colon cancer metastasis. *Gastroenterology* **110**: 1354–1367

Cannistra SA, Kansas GS, NiloffI J, Defranzo B, Kim Y and Ottensmeier C (1993) Binding of ovarian cancer cells to peritoneal mesothelium in vivo is partly mediated by CD44H. *Cancer Res* **53**: 3830–3838

Dohi T, Abe K and Ohshiba S (1989) Immunohistochemical study of carbohydrate antigen expression in gastric carcinoma. *Gastroenterol Jpn* **24**: 239–245

Esaki Y, Hirayama R and Hirokawa K (1990) A comparison of patterns of metastasis in gastric cancer by histologic type and age. *Cancer* **65**: 2086–2090

Ignacio D and Osvaldo L (1981) Patterns of metastases in intestinal and diffuse types of carcinoma of the stomach. *Hum Pathol* **12**: 237–242

Ikeda Y, Mori M, Kajiyama K, Haraguchi Y, Sasaki O and Sugimachi K (1996) Immunohistochemical expression of sialyl Tn, sialyl Lewis$^a$, sialyl Lewis$^{a\text{-}b\text{-}}$ and sialyl Lewis$^x$ in primary tumor and metastatic lymph nodes in human gastric cancer. *J Surg Oncol* **62**: 171–176

Izumi Y, Taniguchi Y, Tsuji T, Smith CW, Nakamori S, Fidler IJ and Irimura T (1995) Characterization of human colon carcinoma variant cells selected for sialyl Le$^x$ carbohydrate antigen: Liver colonization and adhesion to vascular endothelial cells. *Exp Cell Res* **216**: 215–221

Japanese Research Society for Gastric Cancer (1995) *Japanese Classification of Gastric Carcinoma* (First English ed). Kanehara & Co., Ltd, Tokyo.

Jorgensen T, Berner A, Kaalhus O, Tveter KJ, Danielsen HE and Bryne M (1995) Up-regulation of the oligosaccharide sialyl Lewis$^x$: a new prognostic parameter in metastatic prostate cancer. *Cancer Res* **55**: 1817–1819

Lauren P (1965) The two histological main types of gastric carcinoma: diffuse and so-called intestinal type carcinoma. *Acta Path et Microbiol Scandinav* **64**: 31–49

Lowe JB, Stoolman LM, Nair RP, Larsen RD, Berhend TL and Marks RM (1990) ERAM-1-dependent cell adhesion to vascular endothelium determined by a transfected human fucosyltransferase cDNA. *Cell* **63**: 475–484

Ming S (1977) Gastric carcinoma: a clinicopathological classification. *Cancer* **39**: 2475–2485

Mori M, Sakaguchi H, Akazawa K, Tsuneyoshi M, Sueishi K and Sugimachi K (1995) Correlation between metastatic site, histologic type and serum tumour markers of gastric carcinoma. *Hum Pathol* **26**: 504–508

Nagayo T and Komagoe T (1961) Histological studies of gastric mucosal cancer with special reference to relationship of histological pictures between mucosal cancer and the cancer-bearing gastric mucosa. *Gann* **52**: 109–119

Nakagoe T, Fukushima K, Hirota M, Kusano H, Ayabe H, Tomita M and Kamihara S (1993) Immunohistochemical expression of sialyl Lewis$^x$ antigen in relation to survival of patients with colorectal carcinoma. *Cancer* **72**: 2323–2330

Nakamori S, Kameyama M, Imaoka S, Furukawa H, Ishikawa O, Sasaki Y, Kabuto T, Iwanaga T, Matsushita Y and Irimura T (1993) Increased expression of sialyl Lewis$^x$ antigen correlates with poor survival in patients with colorectal carcinoma: clinicopathological and immunohistochemical study. *Cancer Res* **53**: 3632–3637

Nakamori S, Furukawa H, Iwanaga T, Imaoka S, Ishikawa O, Kabuto T, Sasaki Y, Kameyama M, Ishiguro S and Irimura T (1997) Expression of carbohydrate antigen sialyl Le$^a$: a new functional prognostic factor in gastric cancer. *J Clin Oncol*. **15**: 816–825

Narita T, Funahashi H, Satoh Y, Watanabe T, Sakamoto J and Takagi H (1993) Association of expression of blood group-related carbohydrate antigens with prognosis in breast cancer. *Cancer* **71**: 3044–3053

Phillips ML, Nudelman E, Gaeta FCA, Perez M, Singhal AK, Hakomori S and Paulson JC (1990) ELAM-1 mediates cell adhesion by recognition of a carbohydrate ligand, sialyl Le$^x$. *Science* **250**: 1130–1132

Rhomberg W and Gruber U (1989) Liver metastasis in cancer of the stomach and its dependence on the histology of the primary tumor: an autopsy study on 102 cases. *Clin Expl Metastasis* **7**: 585–590

Saitoh O, Wang WL, Lotan R and Fukuda M (1992) Differential glycosylation and cell surface expression of lysosomal membrane glycoproteins in sublines of a human colon cancer exhibiting distinct metastatic potentials. *J Biol Chem* **267**: 5700–5711

Sawada R, Tsuboi S and Fukuda M (1992) Differential E-selectin-dependent adhesion efficiency in sublines of a human colon cancer exhibiting distinct metastatic potentials. *J Biol Chem* **269**: 1425–1431

Takada A, Ohmori K, Takahashi N, Tsuyuoka K, Yago K, Zenita K, Hasegawa A and Kannagi R (1991) Adhesion of human cancer cells to vascular endothelium mediated by a carbohydrate antigen, sialyl Lewis A. *A Biol Biophys Res Comm* **179**: 713–719

Takada A, Ohmori K, Yoneda T, Tsuyuoka K, Hasegawa A, Kiso M and Kannagi R (1993) Contribution of carbohydrate antigens sialyl Lewis A and sialyl Lewis X to adhesion of human cancer cells to vascular endothelium. *Cancer Res* **53**: 354–361

Tsutsumi Y, Onda N and Osamura Y (1990) Victoria blue-hematoxylin and eosin staining: a useful routine stain for demonstration of venous invasion by cancer cells. *J Histotechnol* **13**: 271–274

Ura H, Denno R, Hirata K, Yamaguchi K, Yasoshima T and Shishido T (1997) Close correlation between increased sialyl-Lewis$^x$ expression and metastasis in human gastric carcinoma. *World J Surg* **21**: 773–776

Walz G, Aruffo A, Kolanus W, Bevilacqua M and Seed B (1990) Recognition by ELAM-1 of the sialyl-Le$^x$ determinant on myeloid and tumor cells. *Science* **250**: 1132–1135

Wronkowsky Z, Stemmermann G and Rellahan W (1977) Stomach carcinoma among Hawaiians and Caucasians in Hawaii. *Cancer* **39**: 2310–2316

© 2000 Cancer Research Campaign
